# Cerebrospinal fluid kappa free light chains for the diagnosis of multiple sclerosis: A consensus statement

**DOI:** 10.1177/13524585221134217

**Published:** 2022-12-17

**Authors:** Harald Hegen, Georgina Arrambide, Sharmilee Gnanapavan, Batia Kaplan, Michael Khalil, Ruba Saadeh, Charlotte Teunissen, Hayrettin Tumani, Luisa Maria Villar, Maria Alice V Willrich, Henrik Zetterberg, Florian Deisenhammer

**Affiliations:** Department of Neurology, Medical University of Innsbruck, Innsbruck, Austria; Servei de Neurologia-Neuroimmunologia, Centre d’Esclerosi Múltiple de Catalunya (Cemcat), Vall d’Hebron Hospital Universitari, Universitat Autònoma de Barcelona, Barcelona, Spain; Blizard Institute, Barts and The London School of Medicine and Dentistry, Queen Mary University of London, London, UK; Laboratory of Hematology, Sheba Medical Center, Ramat Gan, Israel; Department of Neurology, Medical University of Graz, Graz, Austria; Department of Laboratory Medicine and Pathology, Mayo Clinic, Rochester, MN, USA/Department of Neurology, Mayo Clinic, Rochester, MN, USA; Neurochemistry Laboratory, Department of Clinical Chemistry, Amsterdam Neuroscience, Program Neuroinflammation, Vrije Universiteit Amsterdam, Amsterdam UMC, Amsterdam, The Netherlands; CSF Laboratory, Department of Neurology, University of Ulm, Ulm, Germany; Biostatistics Unit, Department of Immunology, Hospital Universitario Ramón y Cajal, Madrid, Spain; Department of Laboratory Medicine and Pathology, Mayo Clinic, Rochester, MN, USA; Department of Psychiatry and Neurochemistry, Institute of Neuroscience and Physiology, Sahlgrenska Academy, University of Gothenburg, Gothenburg, Sweden/Clinical Neurochemistry Laboratory, Sahlgrenska University Hospital, Gothenburg, Sweden/Department of Neurodegenerative Disease, UCL Queen Square Institute of Neurology, London, UK/UK Dementia Research Institute at UCL, London, UK/Hong Kong Center for Neurodegenerative Diseases, Hong Kong, China; Department of Neurology, Medical University of Innsbruck, Innsbruck, Austria

**Keywords:** Cerebrospinal fluid, kappa-free light chains, multiple sclerosis, clinically isolated syndrome, diagnosis, disease activity, prediction, biomarker, index, consensus

## Abstract

Cerebrospinal fluid (CSF) analysis is of utmost importance for diagnosis and differential diagnosis of patients with suspected multiple sclerosis (MS). Evidence of intrathecal immunoglobulin G (IgG) synthesis proves the inflammatory nature of the disease, increases diagnostic certainty and substitutes for dissemination in time according to current diagnostic criteria. The gold standard to determine intrathecal IgG synthesis is the detection of CSF-restricted oligoclonal bands (OCBs). However, advances in laboratory methods brought up κ-free light chains (FLCs) as a new biomarker, which are produced in excess over intact immunoglobulins and accumulate in CSF in the case of central nervous system-derived inflammation. Overwhelming evidence showed a high diagnostic accuracy of intrathecal κ-FLC synthesis in MS with sensitivity and specificity of approximately 90% similar to OCB. κ-FLCs have advantages as its detection is fast, easy, cost-effective, reliable, rater-independent and returning quantitative results which might also improve the value of predicting MS disease activity. An international panel of experts in MS and CSF diagnostics developed a consensus of all participants. Six recommendations are given for establishing standard CSF evaluation in patients suspected of having MS. The panel recommended to include intrathecal κ-FLC synthesis in the next revision of MS diagnostic criteria as an additional tool to measure intrathecal immunoglobulin synthesis.

## Introduction

Diagnosis of multiple sclerosis (MS) requires the combination of clinical signs and symptoms with para-clinical findings obtained by magnetic resonance imaging and cerebrospinal fluid (CSF) analysis.^1^ Evidence of intrathecal immunoglobulin G (IgG) synthesis in the CSF, although not specific for MS,^2^ substitutes for dissemination in time according to current diagnostic criteria^1^ and increases diagnostic certainty in the appropriate clinical setting.^3^ The gold standard to prove intrathecal IgG synthesis is the detection of CSF-restricted oligoclonal bands (OCBs).^4^

**Figure 1.**
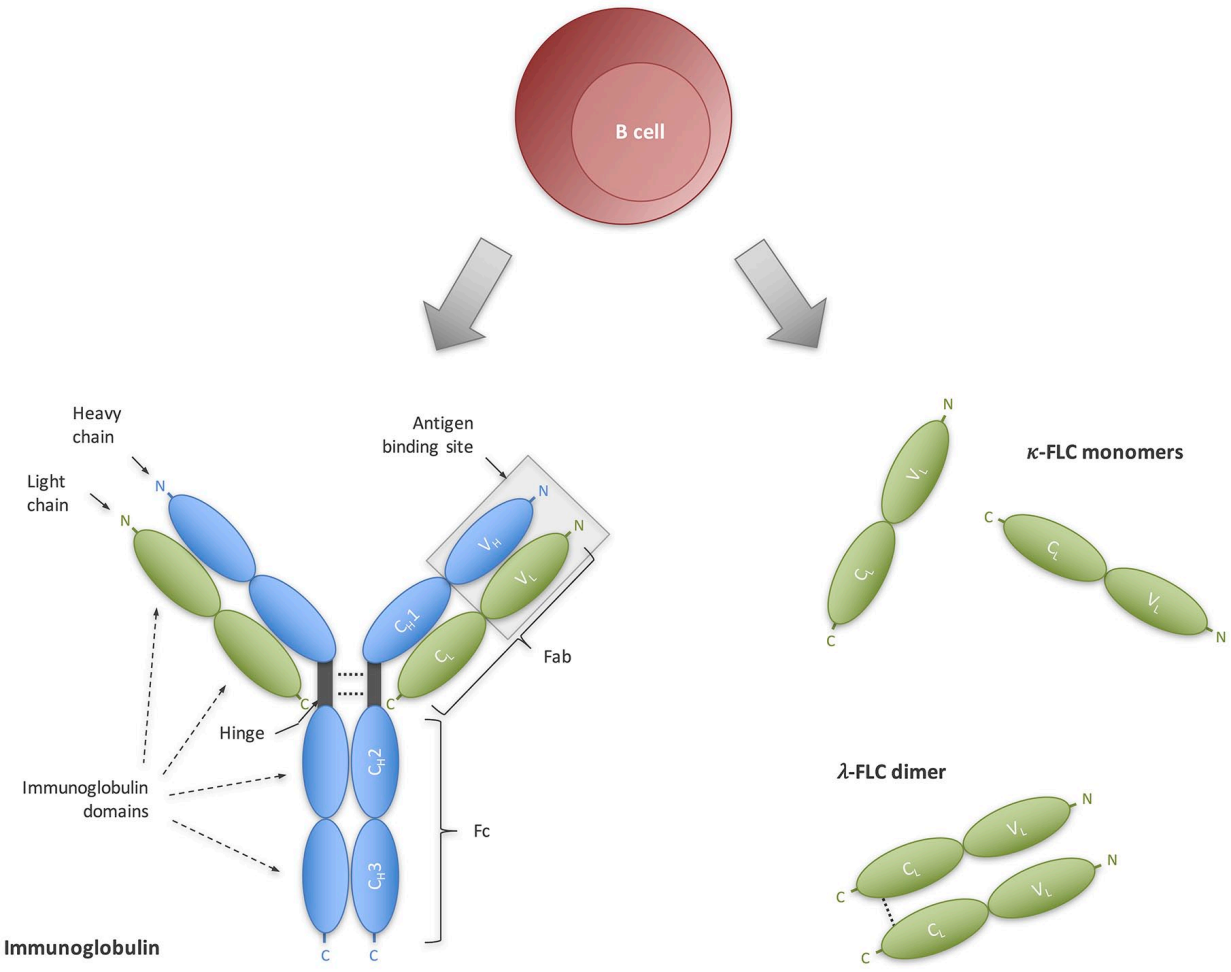
Immunoglobulin FLCs as an emerging biomarker for intrathecal B-cell activity. Terminally differentiated B cells produce (A) intact immunoglobulins that consist of bound light chains (green) and heavy chains (blue), as well as (B) in excess FLCs. Both immunoglobulins and FLC serve as a biomarker for B-cell activity. C_H_: constant heavy chain domain; C_L_: constant light chain domain: Fab; fragment antigen-binding; Fc: fragment crystallizable; FLC: free light chain; V_H_: variable heavy chain domain; V_L_: variable light chain domain.

B cells produce intact immunoglobulins by assembling light chains and heavy chains via disulfide bonds and non-covalent interactions, but B cells also produce light chains in excess of 10%–40% over heavy chains and secrete them as free forms (Figure 1).^5,6^ Similar to immunoglobulins, free light chains (FLCs) accumulate in the CSF in the case of chronic inflammatory diseases of the central nervous system such as MS.^7^ FLCs were discovered long ago; however, their quantitative detection with high sensitivity and, thus, in low-level compartments such as CSF was not possible until technological advances at the beginning of the century.^7^ The breakthrough was achieved by producing detection antibodies directed against unique FLC epitopes.^8^

A multitude of studies have shown a high diagnostic accuracy of the κ-FLC isotype in the CSF to discriminate patients with MS from other neurological diseases;^9^ and the detection of κ-FLC has considerable methodological advantages compared to the detection of OCB.^8,10^ However, a strong consensus on the role of κ-FLC as a biomarker in MS is still lacking. This might be due to a certain heterogeneity between published studies ranging from different patient populations, assays, different κ-FLC measures (e.g. κ-FLC index versus absolute CSF κ-FLC concentration) and cut-off values.

In an effort to evaluate and recommend the type of CSF analysis that yields the greatest diagnostic sensitivity and specificity for the diagnosis of MS considering new technologies in the last two decades, a working group was formed. The aim was to produce a report for neurologists and laboratory medicine specialists on what would be considered as ‘standard’ for the evaluation of CSF in patients with suspected MS and to provide consensus recommendations on the use of κ-FLC in routine diagnostic work-up.

## Methods

In October 2021, an international panel convened in Vienna, Austria, to discuss the use of CSF and, in particular, the applicability of κ-FLC in the CSF for routine diagnostic purposes in patients with suspected MS. The panel was composed of experts in the diagnosis and management of MS patients and/or CSF analysis, including neurologists and laboratory medicine specialists from 11 institutions across eight countries and three continents. All participants are listed as authors of this manuscript. This meeting was endorsed by the European Committee for Treatment and Research in Multiple Sclerosis (ECTRIMS).

The panel presented and discussed data from research published in English about CSF analysis and, in particular, about κ-FLC in patients with clinically isolated syndrome (CIS) and MS. While specific recommendations for the implementation of κ-FLC in the diagnostic process for patients with suspected MS were developed during the meeting, the panel set out to create the whole consensus document afterwards. Furthermore, the panel decided to perform a systematic review and meta-analysis to provide a summary of the diagnostic value of κ-FLC in patients with CIS and MS and to compare its performance to OCB (published elsewhere Hegen et al.^9^).

The consensus document was first drafted by the principal author. The first draft was then circulated to all panellists, who iteratively contributed to the document until an agreement was reached on the final document. For each of the recommendations, a minimum agreement of 90% (i.e. 11 of 12 co-authors) was required. If agreement on a recommendation was not achieved, it was discussed again, and a new proposal re-circulated, until a final agreement was achieved.

### Routine CSF panel in MS diagnostic work-up

The full spectrum of routine CSF parameters including white blood cell (WBC) count, differential cell profile (assessed, e.g. by inspection of CSF cytology), albumin quotient (*Q*_alb_) and intrathecal Ig synthesis contributes to the diagnosis of MS and the exclusion of other causes of CNS inflammation mimicking MS, for example, vasculitis, chronic infection or other acquired demyelinating disorders, such as neuromyelitis optica spectrum disorders (NMOSD) and myelin oligodendrocyte glycoprotein-associated disorders (MOGAD).^4^ A recent, comprehensive study on routine CSF parameters in more than 500 patients with CIS and MS applying the McDonald criteria 2017 revealed that approximately 50% of patients show CSF pleocytosis with a WBC count up to 40/µL (95th percentile), that lymphocytes followed by monocytes are the predominant cell type in CSF cytology, that the blood-CSF-barrier function is abnormal in less than 10% of cases with *Q*_alb_ up to approximately 10 (95th percentile), and that intrathecal IgG synthesis as determined by CSF-restricted OCB are present in up to 95% of patients.^11^ CSF findings slightly differ between the different MS disease courses; for example, patients with progressive MS show lower WBC counts and less frequently CSF pleocytosis; and CSF-restricted OCBs are found in approximately 80% of CIS patients but in up to 95% of MS patients^10^ with lower prevalence in areas of lower geographic latitude.^12^ Relevant differential diagnoses frequently show higher WBC counts, different WBC subpopulations (e.g. relevant percentages of neutrophils) and higher *Q*_alb_ and OCB infrequently (only up to 10%–20%).^13,14^ For details on CSF collection and analysis, we refer to previously published consensus guidelines.^2,4,15^

#### Consensus statement 1

Neurologists need to consider the results of all tests performed as part of the CSF panel (e.g. white blood cell count, differential cell profile, albumin quotient, intrathecal Ig synthesis, CSF/serum glucose ratio or CSF lactate), which should be interpreted in the context of clinical and imaging findings.

### Intrathecal immunoglobulin synthesis

Different methods are available for the detection of intrathecal immunoglobulin synthesis in patients with suspected MS, each with certain strengths and limitations.

**Figure 2.**
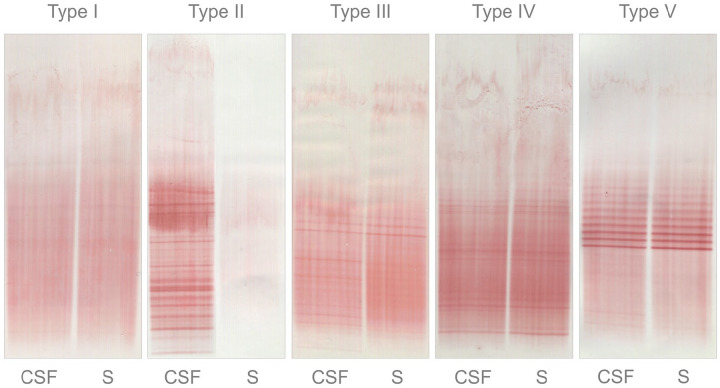
Pattern of oligoclonal IgG bands. Type I No bands in CSF and serum. Type II OCBs present in CSF without corresponding bands in serum. Type III OCBs present in both CSF and serum, with additional bands present in CSF. Type IV OCBs present in CSF, which are identical to those in serum ( ‘mirror pattern’). Type V bands present in CSF, which are identical to those in serum ( ‘ladder pattern’). CSF: cerebrospinal fluid; OCBs: oligoclonal bands; S: serum.

1. Quantitative intrathecal IgG synthesis: The concentration of total IgG in CSF and serum are determined followed by the calculation of certain formulae such as IgG index,^16^ Reiber^17^ or Auer et al.^18^ formulae referring patients’ individual values to a predefined upper normal limit. This approach shows a moderate diagnostic sensitivity of approximately 70% in MS patients.^11^
2. Qualitative intrathecal IgG synthesis: The detection of oligoclonal IgG bands (OCB) by isoelectric focussing (IEF) followed by immuno-detection is currently the gold standard method.^1,4^ This technique compares paired CSF and serum samples of each individual patient. Intrathecal IgG synthesis is demonstrated if OCBs are present in CSF without corresponding bands in the serum (patterns II and III) (Figure 2).^4^ This method shows high diagnostic sensitivity and specificity both of approximately 90%,^19^ however, provides the qualitative determination of an intrathecal IgG synthesis (i.e. positive or negative).^4^
3. Quantitative intrathecal κ-FLC synthesis: κ-FLC concentrations are measured in CSF and serum followed by calculation of either the κ-FLC index or an intrathecal κ-FLC fraction (IF_κ-FLC_), where again patients’ individual values are compared to a predefined upper normal limit. An overview of the different κ-FLC measures investigated in MS is provided in Table 1. Certain aspects, for example, considering only absolute CSF κ-FLC concentrations, are addressed below.

**Table 1.** Various κ-FLC parameters investigated in MS.

■ CSF κ-FLC concentration ■ $Q_{k-\mathrm{FLC}}=\frac{\kappa -{\mathrm{FLC}}_{CSF}}{\kappa -{\mathrm{FLC}}_{Serum}}$ ■ $\kappa -\mathrm{FLC}\;\mathrm{index}=\frac{\frac{\kappa -{\mathrm{FLC}}_{CSF}}{\kappa -{\mathrm{FLC}}_{Serum}}}{\frac{{\mathrm{Albumin}}_{CSF}}{{\mathrm{Albumin}}_{Serum}}}$ ■ IF _κ-FLC_ with different underlying formulae to determine the *Q*_alb_-dependent upper reference limit (*Q*_lim κ-FLC_) and, thus, the limit to define IF_κ-FLC_ - Presslauer et al.:^20^ $Q_{\lim-FLC}=0.9358\times Q_{alb}^{0.6687}$ - Hegen et al.:^21^ $Q_{\lim-FLC}=3.1276\;\times Q_{alb}^{0.8001}$ - Senel et al.:^22^ $Q_{\lim-FLC}=9.5+2.08\times Q_{alb}$ - Reiber et al.:^23^ $Q_{\lim-FLC}=3.27\times {\left(Q_{alb}^{2}+33\right)}^{0.5}-8.2\times 10^{-3}$

CSF: cerebrospinal fluid; FLC: free light chain; IF: intrathecal fraction; *Q*_alb_: CSF/serum albumin quotient.

### Diagnostic accuracy of intrathecal κ-FLC synthesis in patients with CIS and MS

A systematic review and meta-analysis summarized the evidence on the diagnostic accuracy of intrathecal κ-FLC synthesis to discriminate patients with CIS and MS from other neurological diseases and compared its performance to OCB.^9^

Most evidence exists for κ-FLC index with 32 studies performed on approximately 3300 CIS/MS patients and 5800 control subjects. The κ-FLC index showed a diagnostic sensitivity ranging from 52% to 100% (weighted average: 88%) and specificity from 69% to 100% (89%). OCB had a diagnostic sensitivity of 37% to 100% (85%) and a specificity of 74% to 100% (92%). The comparison of these two parameters by bivariate mixed model – considering between-study and within-study heterogeneity and having a statistical power of 99% – clearly showed that the diagnostic accuracy of κ-FLC index and OCB are similar.^9^

The other parameters previously used to determine intrathecal κ-FLC, for example, IF_κ-FLC_, or the CSF κ-FLC concentration, also achieved a diagnostic accuracy, which was similar to OCB. However, due to the low number of studies the statistical power for these comparisons was smaller than 80% and, thus, insufficient to interpret non-statistically significant results with a small enough Type II error. For detailed analyses please refer to Hegen et al.^9^

#### Consensus statement 2

The single most informative analysis in MS, although not disease-specific, is the assessment of intrathecal immunoglobulin synthesis, either by qualitative detection of a CSF-unique IgG fraction (using IEF followed by immunodetection), or a quantitative CSF κ-FLC fraction (using nephelometry or turbidimetry).

### Different parameters to determine intrathecal κ-FLC synthesis

κ-FLC in the CSF – similar to immunoglobulins or other proteins – originate either from the blood by diffusion across the blood-CSF-barrier, or by intra-thecal production under pathological conditions.^24^ Conceptually, it seems necessary to determine the locally synthesized κ-FLC fraction separate from the blood-derived fraction as done for total IgG. The majority of studies used the κ-FLC index^22,25–55^ or IF_κ-FLC_.^22,29,34,38–41,43,48,55–58^ Both approaches consider *Q*_alb_ which is an established marker of the blood-CSF-barrier function^2^ and correct for the absolute serum κ-FLC concentration. Few studies used *Q*_κ-FLC._^22,37,39,51^ Other authors determined the absolute CSF κ-FLC concentrations only.^33,34,39,41,48,50,51,59–61^ As the intra-thecal originated κ-FLC fraction is greater than 80% in most CIS/MS patients,^23,29^ one might argue that the contribution of blood-derived κ-FLC to the total CSF κ-FLC concentration is negligible in cases with intrathecal synthesis. One study reported that around 15% of CIS/MS patients showed even higher absolute κ-FLC concentrations in CSF than in serum that proves an intrathecal synthesis per se.^62^

The above-mentioned meta-analysis compared the performance of κ-FLC index, IF_κ-FLC_ and CSF κ-FLC concentration and observed a similar diagnostic accuracy. However, the statistical power was below 80% and, thus, insufficient to interpret non-statistically significant results. Therefore, the superiority of, for example, κ-FLC index over CSF κ-FLC concentration cannot be excluded.^9^ There is evidence of one recent study that specifically addressed this question, separated patients into low and high CSF κ-FLC categories and observed that κ-FLC index, IF_κ-FLC_, *Q*_κ-FLC_ and CSF κ-FLC concentration showed similar diagnostic performance in the high category, but not in the low category with the inferiority of CSF κ-FLC and to some extent also of *Q*_κ-FLC_.^62^ One might conclude that the impact of serum κ-FLC and *Q*_alb_ is indeed negligible in patients with high intrathecal κ-FLC synthesis, but probably not in patients with only low or modest intrathecal κ-FLC production. A very recent large multicenter study including more than 1600 patients also reported that κ-FLC index and IF_κ-FLC_ performed slightly better than absolute CSF κ-FLC concentration.^63^ Further studies are required to compare the different κ-FLC measures in patients with varying degrees of intrathecal B-cell activity, varying blood-CSF-barrier function and varying serum κ-FLC concentrations, as the impact of grossly elevated serum FLC levels or elevated *Q*_alb_ has not been sufficiently investigated. This might be of interest also in terms of differential diagnosis, as, for example, NMOSD sometimes shows considerable blood-CSF-barrier dysfunction,^13^ which might lead to higher diffusion of κ-FLC from the blood into CSF and possibly to false-positive results if only absolute CSF κ-FLC concentrations are measured.

#### Consensus statement 3

Methods considering CSF/serum κ-FLC concentration and CSF/serum albumin quotient, for example, the κ-FLC index, show a good overall agreement with CSF κ-FLC concentrations, but seem to be superior in cases with low or modest intrathecal κ-FLC production.

### Analytic aspects of κ-FLC determination

For the determination of κ-FLC in CSF and serum, nephelometry or turbidimetry is widely used and have, compared to, for example, enzyme-linked immunoassays, the advantage that automated single measurements are feasible. It was the perception of the panel that nephelometry or turbidimetry is widely accessible. With regard to the assays, κ-FLC can be measured by use of either polyclonal (Freelite, The Binding Site, Birmingham, UK)^8^ or monoclonal (N Latex, Siemens, Erlangen, Germany)^10^ detection antibodies.

### Monoclonal versus polyclonal detection antibody assay on different platforms

Polyclonal assays contain anti-κ-FLC antibodies that are raised in sheep after immunization with a pool of human κ-FLC followed by an adsorption step against intact immunoglobulin (containing bound light chains), so that only κ-FLC-specific antibodies remain in the final antisera.^8^ In contrast, monoclonal assays contain a cocktail of multiple monoclonal antibodies which are each produced in hybrid cell lines and finally mixed together.^10^ Due to these differences in the production, certain characteristics in assay performance arise, for example, a higher lot-to-lot variation in polyclonal assays.^64,65^

There are a few studies that compared the polyclonal (Freelite) and monoclonal (N Latex) assay in serum and found a moderate agreement as determined by correlation analyses (with a coefficient of > 0.9) or Passing–Bablok regression (with a slope between 0.74 and 0.99)^10,66–68^ as well as by concordance rates (sample classification, ranging from 77 up to 91%).^10,67,68^ However, these studies contained samples from patients with, for example, monoclonal gammopathy covering κ-FLC measurements ranging up to thousands mg/L.^67,68^ The differences observed between the methods occurred mainly at the upper end of the analytical range.^10,67,68^ These extremely elevated concentrations found in the serum of monoclonal gammopathy patients are not relevant in MS patients without these co-morbidities.

Scarce evidence on assay comparison exists for absolute CSF κ-FLC concentration (Passing–Bablok regression, slope of 0.85)^66^ and κ-FLC index (Passing–Bablok regression, slope of 0.94) with moderate agreement.^68^

The choice of the platform has an impact on serum κ-FLC measurement^68^ and reference intervals^69^; however, there are no studies addressing the impact on CSF κ-FLC.

Considering the information provided on the platform and assay-specific variations, it is plausible to assume that κ-FLC index might be less prone to laboratory variations compared to absolute CSF κ-FLC concentration (due to the use of CSF/serum κ-FLC ratio resulting in a dimensionless variable). The above-mentioned meta-analysis did not show a statistically significant difference between κ-FLC index across the methods employing monoclonal or polyclonal antibodies as reagents in multiple platforms clinically available.^9^ However, this has to be investigated by further research.

### Pre-analytics and robustness

κ-FLC in the CSF are by far less susceptible to blood contamination, which can occur due to traumatic lumbar puncture, as compared to other CSF proteins, for example, immunoglobulins. A study showed that even in case of a blood contamination that led to false-positive intrathecal IgG synthesis in almost 90% of patients as determined by the Reiber^70^ formula, intrathecal κ-FLC synthesis was still not affected. An explanation is probably the small molecular size of κ-FLC of approximately 24 kDa compared to the larger size of immunoglobulins, for example, of 150 kDa for IgG.^71,72^ Smaller molecules show a higher CSF/serum ratio, and in the case of artificial blood contamination, the relative increase of CSF concentration is lower. With regard to OCB, blood contamination reduces the chance of detecting faint bands and might lead to false negative results.

Free light chains are stable in serum samples frozen at −20°C for a storage duration of at least 1 year. Whether thereafter is a relevant change has not been investigated yet.^73^ Studies investigating CSF stability are missing too.

Patient-related factors might impact κ-FLC concentration. κ-FLC in serum might depend on renal clearance, that is, higher serum FLC concentrations were found in older patients with decreased renal function.^74^ Most studies on the biological variation of serum κ-FLC apart from an underlying disease revealed a small within-subject variation of <10%.^75,76^ Whether this small within-subject variation can also be extrapolated to CSF levels in MS has to be determined. Recently, it has been shown that high-dose corticosteroids resulted in lower serum FLC concentrations; however, CSF levels and, more importantly, the FLC index were not affected.^77^

### Cut-off points to determine intrathecal κ-FLC synthesis

Cut-off points might depend on the clinical question, that is, whether an upper reference limit is determined in a non-inflammatory control population,^78^ or if a cut-off is determined to discriminate patients with MS from other inflammatory neurological diseases. Cut-off points might also vary depending on whether the focus is to increase diagnostic sensitivity or diagnostic specificity.^66^ The impact of laboratory methods on κ-FLC measurements, which again might influence cut-off points, has been discussed above.

The vast majority of studies using κ-FLC index, CSF κ-FLC concentration, *Q*_κ-FLC_ as well as different formulae to define *Q*_lim κ-FLC_^20,22,23^ for calculation of IF_κ-FLC_ compared CIS/MS patients to heterogeneous control populations, and only the minority used pure non-inflammatory disease controls. Furthermore, most of these studies applied a discriminatory cut-off rather than a cut-off that maximizes either diagnostic sensitivity or diagnostic specificity.^9^

For the κ-FLC index, reported cut-off values ranged from 2.4 to 20. In the above-mentioned meta-analysis, a mean cut-off of 6.1 could be determined.^9^ Even though this cut-off is in line with those identified by several large – partly multicenter – studies,^29,33,45,47^ it has to be clearly stated that this was an exploratory analysis.

For CSF κ-FLC concentration, a mean discriminatory cut-off to differentiate CIS/MS patients from controls at 0.96 mg/L was observed. Here again, this cut-off is the result of exploratory analysis and is based only on a limited number of studies.^9^ With regard to the different non-linear formulae,^20,22,23^ the small number of studies did not allow any between-study comparisons.^9^ There is only one study that compared the performance of all three formulae within an independent cohort reporting similar diagnostic sensitivities ranging from 96% to 98% in MS patients and 40% to 44% in CIS patients.^40^ A comparison in terms of specificity is still lacking. Due to the small number of studies, no cut-off for *Q*_FLC_ could be determined.^9^ At this point, we would like to state that besides laboratory variations, also handling non-detectable CSF values might have an impact on κ-FLC index values. This means that reported cut-off values might be biased by this issue. For a detailed discussion, we refer to Hegen et al.^9^

#### Consensus statement 4

There is extensive data on quite similar cut-off values for κ-FLC index. However, multicenter studies using different platforms and assays should be performed to definitively confirm these cut-offs, and certified reference materials should be developed.

### Will κ-FLC replace OCB detection?

Determination of intrathecal κ-FLC synthesis and CSF-restricted OCB has certain strengths and limitations (Table 2).

**Table 2.** Comparison of CSF-restricted OCB and intrathecal κ-FLC synthesis for diagnosis of MS.

κ**-FLC synthesis** (e.g. κ-FLC index) reflects intrathecal IgG synthesis, but also IgA and IgM synthesis PROs ■ High diagnostic sensitivity and specificity ■ High stability in CSF and serum ■ Robustness (e.g., blood contamination) ■ Easy and fast method ■ Labour- and cost-effective ■ Quantitative result ■ Interpretation of results is rater-independent CONTRA ■ Does not differentiate between IgG clonality and distinct IgG synthesis patterns	**Oligoclonal bands** detect intrathecal IgG synthesis PROs ■ High diagnostic sensitivity and specificity ■ High stability in CSF and serum ■ Robustness ■ Detection of IgG clonality in CSF and serum compartments (poly-, oligo-, monoclonal) ■ Differentiation of 2 distinct patterns of intrathecal IgG synthesis CONTRA ■ Time-consuming and technically demanding method ■ Labour-intensive and costly ■ Qualitative result (i.e. either positive or negative) ■ Interpretation of results is rater-dependent

CSF: cerebrospinal fluid; FLC: free light chain; Ig: immunoglobulin; OCB: oligoclonal bands.

As outlined above, the intrathecal κ-FLC synthesis shows a high diagnostic accuracy to discriminate patients with CIS and MS from other neurological diseases with a sensitivity and specificity of approximately 90%, similar to OCB.^9^ κ-FLC is measured by nephelometry or turbidimetry which is – in contrast to the detection of OCB – an easy, reliable, labour-saving, cost-efficient and rater-independent method.^8,10^ The intrathecal κ-FLC synthesis, for example, by determination of the κ-FLC index, returns a metric result,^29^ while OCB status is dichotomous returning either a positive or negative result.^4^ The quantitative result of κ-FLC might gain additional utility in the prediction of disease activity in early MS (discussed below).

However, it cannot be differentiated whether increased κ-FLC measures are the consequence of an intrathecal IgA, IgM and/ or IgG synthesis. CSF κ-FLC levels do not provide information on the clonality of immunoglobulin production and differentiation between systemic inflammation with an additional intrathecal inflammation (OCB Pattern III), or an isolated intrathecal inflammation (OCB Pattern II) is not possible. This additional information provided by OCB can be helpful in some clinical situations, for example, in patients with monoclonal gammopathies and suspected CNS involvement (i.e. Bing Neel syndrome),^79,80^ or in patients with the CNS involvement of systemic diseases (e.g. neurosarcoidosis and systemic lupus erythematosus, which show pattern III more frequently than MS patients).^81^

Determination of κ-FLC in the CSF and serum requires a sample volume of at least 200 µL (due to the dead volume in the cuvette placed in the nephelometer or turbidimeter). Even though OCB testing requires only a minimum of approximately 10–20 µL (placed on the gel for IEF),^19,82^ prior determination of IgG concentration is recommended so that the appropriate dilution of samples can be performed and, thus, the same amount of IgG applied for the IEF run.

### Reflex approach

**Figure 3.**
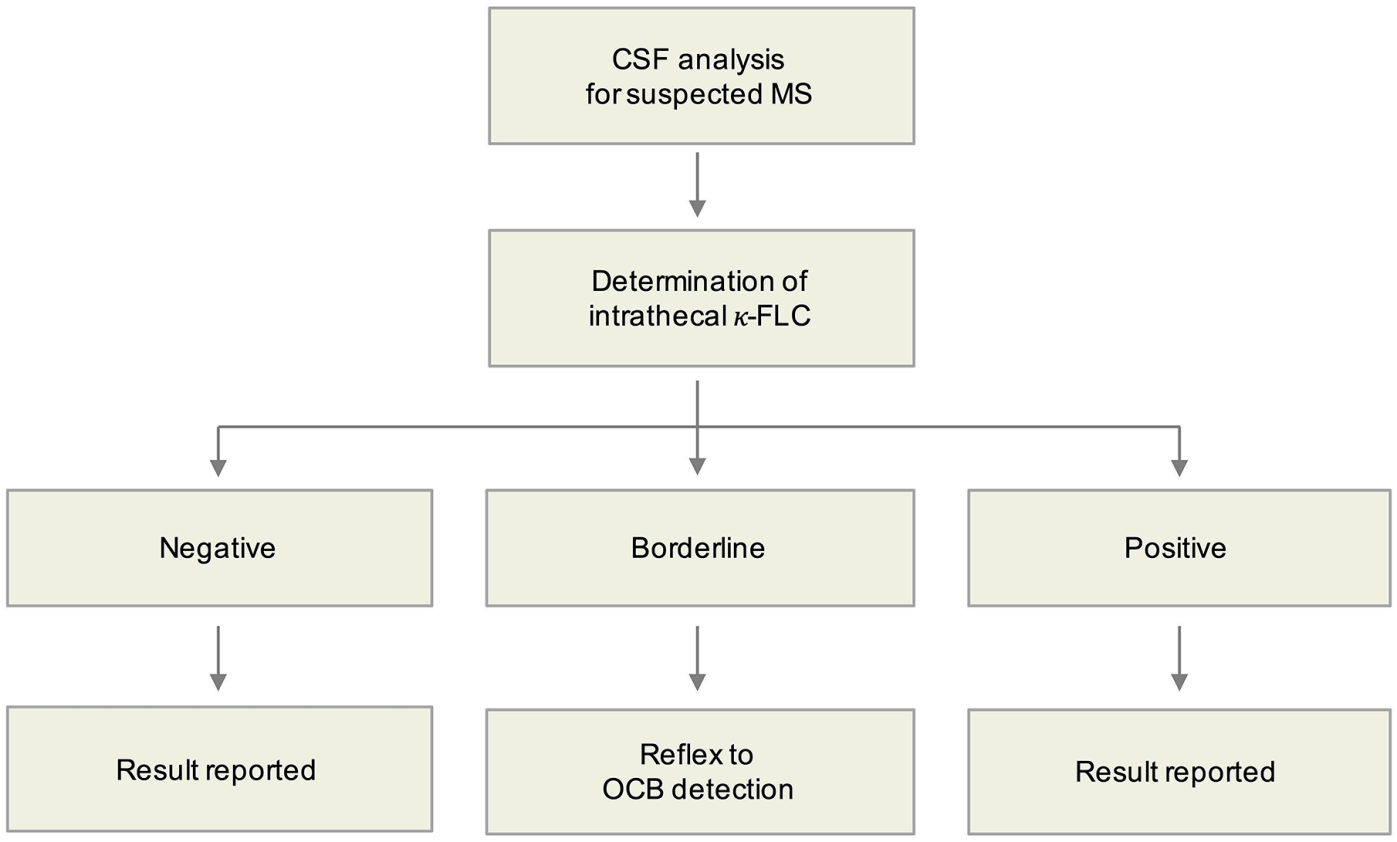
Strategy reflex algorithm. FLC: free light chain; MS: multiple sclerosis; OCB: oligoclonal bands.

Determination of intrathecal κ-FLC synthesis might be used as a first-line screening test in MS. The reflex approach applies two cut-off points and reports results in case of clearly negative or clearly positive values. In the case of values between the two cut-off points ( ‘grey zone’), OCB detection should follow as a second step (Figure 3).

The low cut-off should ensure that patients with a negative result have indeed no signs of intrathecal B-cell activity, while the second higher cut-off should unequivocally identify patients with intrathecal B-cell activity. While in the majority of CIS and MS patients the extent of intrathecal κ-FLC synthesis is high and the interpretation as a positive result clear, in case of low positives, several issues should be considered including analytic and biological variation. The low cut-off will label a certain proportion of samples as κ-FLC-positive in the absence of OCB in CSF. Some cases will be explained by intrathecal IgA or IgM synthesis.^21^ Discrepancies between intrathecal κ-FLC synthesis and OCB might also arise from different cut-offs defining OCB positivity, which is different numbers of CSF-restricted bands.^19^ Of interest, there are studies reporting that relevant differential diagnoses of MS, for example, NMOSD, show lower κ-FLC levels which might fall into a grey zone (e.g. κ-FLC index of approximately 90 in MS, 20 in NMOSD and 4 in controls).^52^ Further studies are needed to investigate patients with low intrathecal κ-FLC levels.

Studies evaluating the financial aspect of a reflex approach found the sequential use of κ-FLC as a screening test and if needed OCB as a confirmation test being less expensive, in terms of reagents, material and personnel as compared to OCB detection. Further advantages of the reflex approach still include a reduction in turn-around times and faster reporting of results.^30,34,60^

However, as evidence defining the ‘grey zone’ is still lacking, one might suggest, at least for the κ-FLC index, using the lowest and highest cut-off points that have been published, that is 2.4 and 20 for the κ-FLC index, respectively.^9^ These cut-off points might also be the rationale for further studies.

#### Consensus statement 5

If results on intrathecal κ-FLC are borderline, an evaluation by OCB can help to clarify the presence of an intrathecal immunoglobulin synthesis, or vice versa. However, until evidence defining borderline intrathecal κ-FLC synthesis is established, the combination of both tests might be the best option at this moment.

### Further research issues

#### Prognostic value of intrathecal κ-FLC synthesis

There are only a few studies on the predictive value of κ-FLC index in MS. An overview is given in Hegen et al.^7^ The majority of studies reported that intrathecal κ-FLC synthesis is associated with conversion from CIS to MS^28,35,40,53,83^ and that the extent of intrathecal inflammation as reflected by the κ-FLC index predicted the time to conversion to MS as well as disability progression.^83,84^ Two recent studies showed in a multivariate approach considering other already known risk factors such as baseline MRI lesions that in patients with a first CNS demyelinating event, high κ-FLC index is an independent risk factor for early second clinical attack^54,85^ and fulfilment of 2017 McDonald criteria.^85^ These findings fit to previous studies that reported a predictive value of IgG index^86,87^ and the number of OCB,^87–89^ that is, the extent of intrathecal inflammation, with future MS disease activity. In clinical practice, intrathecal κ-FLC synthesis might serve as a predictive biomarker in MS, that is, it could – together with other surrogate markers such as MRI – identify patients in need of early, highly efficacious disease-modifying treatment and, thus, facilitate treatment decision-making.

#### Consensus statement 6

Determination of intrathecal κ-FLC synthesis should be included into the next revision of MS diagnostic criteria as an additional tool to measure intrathecal immunoglobulin synthesis (Table 3).

**Table 3.** Six recommendations (commandments) for CSF κ-FLC detection.

1. Neurologists need to consider the results of all tests performed as part of the CSF panel (e.g. white blood cell count, differential cell profile, albumin quotient, intrathecal Ig synthesis, CSF/serum glucose ratio or CSF lactate), which should be interpreted in the context of clinical and imaging findings. 2. The single most informative analysis in MS, although not disease-specific, is the assessment of intrathecal immunoglobulin synthesis, either by qualitative detection of a CSF-unique IgG fraction (using IEF followed by immunodetection), or a quantitative CSF-unique κ-FLC fraction (using nephelometry or turbidimetry). 3. Methods considering CSF/serum κ-FLC concentration and CSF/ serum albumin quotient, for example, the κ-FLC index, show a good overall agreement with CSF κ-FLC concentrations, but seem to be superior in cases with low or modest intrathecal κ-FLC production. 4. There is extensive data on quite similar cut-off values for κ-FLC index. However, multicenter studies using different platforms and assays should be performed to definitively confirm these cut-offs, and certified reference materials should be developed. 5. If results on intrathecal κ-FLC are borderline, an evaluation by OCB can help to clarify the presence of an intrathecal immunoglobulin synthesis, or vice versa. However, until evidence defining borderline intrathecal κ-FLC synthesis is established, the combination of both tests might be the best option at this moment. 6. Determination of intrathecal κ-FLC synthesis should be included in the next revision of MS diagnostic criteria as an additional tool to measure intrathecal immunoglobulin synthesis.

CSF: cerebrospinal fluid; FLC: free light chain; IEF: isoelectric focussing; MS: multiple sclerosis; OCB: oligoclonal bands.
